# Chinese herbal formula Huayu-Qiangshen-Tongbi decoction ameliorates rheumatoid arthritis through enhancing the release of exosomal miR-125b-5p derived from adipose-derived stem cells by CD63

**DOI:** 10.1186/s40659-025-00628-z

**Published:** 2025-07-10

**Authors:** Wu Xiao-dong, Liang You-bang, Niu Yun-bao, Yang Yi-hong, Huang Wen-zhi, Wang Mao-jie, Mei Li-yan, Gao Kai-xin, Huang Run-yue, Chen Xiu-min

**Affiliations:** 1 State Key Laboratory of Traditional Chinese Medicine Syndrome, The Second Affiliated Hospital of Guangzhou, University of Chinese Medicine, (Guangdong Provincial Hospital of Chinese Medicine), Guangzhou, China; 2 Second Clinical Medical College, Guangzhou University of Chinese Medicine, Guangzhou, Guangdong China; 3 Guangdong-Hong Kong-Macau Joint Lab on Chinese Medicine and Immune Disease Research, Guangzhou, China; 4 Guangdong Provincial Key Laboratory of Clinical Research on Traditional, Chinese Medicine Syndrome, Guangzhou, China; 5 Guangdong Provincial Key Laboratory of Chinese Medicine for Prevention and Treatment of Refractory Chronic Diseases, Guangzhou, China; 6 The Second Affiliated Hospital, Guangzhou University of Chinese Medicine (Guangdong Provincial Hospital of Chinese Medicine), Guangzhou, 510120 China

**Keywords:** Arthritis, Rheumatoid, Huayu-Qiangshen-Tongbi decoction, Exosomal miR-125b-5p, Adipose-derived stem cells, CD63 protein, Human

## Abstract

**Background:**

Rheumatoid arthritis (RA) is a chronic and multifactorial inflammatory disease inducing damages in joints and extra-articular organs. Our previous study has revealed that Huayu-Qiangshen-Tongbi decoction (HQT) ameliorates RA through upregulating microRNA (miRNA) miR-125b-5p to suppress inflammation in rheumatoid fibroblast-like synoviocytes (FLSs). However, the mechanism of HQT increasing miR-125b-5p level in FLSs remains unclear. It has been reported that exosomal miR-125b-5p derived from adipose-derived stem cells (ADSCs) could ameliorate various diseases, yet the effect of exosomal miR-125b-5p derived from ADSCs on FLSs in RA under HQT treatment is largely unknown. The aim is to investigate whether HQT upregulated miR-125b-5p in FLSs through modifying exosomal miR-125b-5p derived from ASCs.

**Methods:**

Here, HQT-containing serum was prepared, co-culture of human FLS MH7A cells with ADSCs was performed, gene knockdown in ADSCs was assessed by short hairpin RNA (shRNA), protein degradation was identified after cycloheximide (CHX) treatment and ADSC-derived exosomes were collected to incubate MH7A cells and inject into RA rat model.

**Results:**

HQT elevates lipopolysaccharide (LPS)-reduced miR-125b-5p level in FLSs by enhancing the secretion of exosomal miR-125b-5p derived from ADSCs. Besides, HQT attenuates inflammation of FLSs in RA by exosomal miR-125b-5p derived from ADSCs in vitro and in vivo. Mechanistically, HQT suppresses CD63 degradation in ADSCs to facilitate the release of exosomal miR-125b-5p derived from ADSCs.

**Conclusion:**

In summary, these findings reveal the mechanism of HQT elevating miR-125b-5p expression in FLSs and provide novel therapeutic strategy for RA treatment.

**Supplementary Information:**

The online version contains supplementary material available at 10.1186/s40659-025-00628-z.

## Introduction

Rheumatoid arthritis (RA) is a chronic and multifactorial inflammatory disease [1, 2]. It has been considered as a syndrome inducing damages in joints and extra-articular organs, such as the lung, kidney, heart, eye, nervous system and digestive system [3, 4]. Nowadays, the global incidence of RA is 0.5-1%, which is more common in women [1]. Exploration of effective drugs for RA is urgently needed.

As a Chinese medicine compound, Huayu-Qiangshen-Tongbi decoction (HQT) developed by The Second Affiliated Hospital of Guangzhou University of Chinese Medicine has been approved to exert a potent therapeutic effect on RA with less adverse effects compared to methotrexate (MTX) [5, 6]. Toll-like receptors (TLRs) are considered as the most crucial immunological mediators in human [7, 8], and inhibiting TLR-mediated NF-kappa-B (NF-κB) could alleviate RA [9]. Similarly, our previous study has revealed that HQT ameliorates RA through upregulating microRNA (miRNA) miR-125b-5p to suppress inflammation induced by NF-κB in rheumatoid fibroblast-like synoviocytes (FLSs) [10]. However, the mechanism of HQT elevating miR-125b-5p expression to block NF-κB pathway in FLSs remains unknown.

Adipose tissue is one of the largest endocrine organs [11], and adipose-derived stem cells (ADSCs) mediate remote communication and collaborative regulation between adipose tissue and other tissues in various diseases by exosomal miRNAs. For instance, exosomal miR-215-5p derived from ADSCs alleviates epithelial-mesenchymal transition of podocytes in diabetic nephropathy by suppressing zinc finger E-box binding homeobox 2 (ZEB2) [12]. Besides, exosomal miR-21 derived from ADSCs facilitates macrophage M2 polarization to enhance angiogenesis in ischemic hindlimb [13]. Moreover importantly, exosomal miR-125-5p derived from ADSCs suppresses the ferroptosis of pulmonary microvascular endothelial cells to attenuate sepsis-induced lung injury [14]. In addition, exosomal miR-125b-5p from adipose-derived mesenchymal stem cells promotes ischemic muscle reparation by targeting alkaline ceramidase 2 [15]. Nevertheless, the effect of Chinese medicine (TCM) on exosomal miR-125b-5p derived from ADSCs and the role exosomal miR-125b-5p derived from ADSCs in FLSs during RA development have not been reported.

The interplay of TCM and exosomes has been a research hot pot as TCM formulas or compounds isolated from TCM exert effects on various diseases through regulating exosomal miRNAs. It has been indicated that Jian-Pi-Yi-Shen formula attenuates adenine-induced chronic kidney disease by modifying circulating exosomal miRNAs [16]. Besides, shikonin prohibits breast cancer cell proliferation by decreasing tumor-derived exosomal miR-128 [17]. CD63 molecule (CD63) is a recognized exosome maker, which contributes to exosome formation, cargo sorting, exosome secretion and cell-specific entry [18–20]. Therefore, modification of CD63 should affect exosome formation, secretion and transfer of cargo in exosomes [19, 21, 22]. However, the effect of TCM on CD63 is largely unknown.

Therefore, the primary aim of current study was to investigate whether HQT upregulated miR-125b-5p in FLSs through modifying exosomal miR-125b-5p derived from ASCs by CD63.

## Materials and methods

### Cell culture

Human ADSCs and human FLS MH7A cells were obtained from the Cell Bank at the Chinese Academy of Sciences (Shanghai, China). Besides, human ADSCs were cultured with Human Adipose-derived Stem Cell Complete Culture Medium (#CM-H205, Procell, Wuhan, Hubei, China) supplemented with 10% fetal bovine serum (FBS) (#SH30087.01, HyClone, Logan, UT, USA), 100U/ml penicillin and 100 µg/mL streptomycin (#SH30010, HyClone) at 37 °C with 5% CO_2_. In addition, MH7A cells were cultured in DMEM (#SH30022.01B, HyClone) containing 10% FBS (#SH30087.01, HyClone), 100U/ml penicillin and 100 µg/mL streptomycin (#SH30010, HyClone) at 37 °C with 5% CO_2_ as previously described [10].

### Preparation of HQT-containing serum

Twelve Wistar rats (male, 8 weeks) purchased from Model Animal Research Center of Nanjing University (Nanjing, Jiangsu, China) were randomly divided into control group and HQT group on average. Rats of HQT group were given 37.6 g/kg HQT from The Second Affiliated Hospital of Guangzhou University of Chinese Medicine by oral gavage once a day for 7 days, while rats of negative control group were given equal volume of physiological saline by oral gavage. During oral gavage, rats were fed with normal diet. Two hours after the last gastric perfusion, abdominal aortic blood of rats was collected into 15mL centrifuge tubes followed by standing at room temperature (RT) for 1 h. Then tubes were centrifuged at 3600 r/minute for 10 min and the supernatant was collected into new centrifuge tubes to inactivate at 56℃ for 0.5 h. Next, the HQT-containing serum was stored at -80℃.

### Cell treatments

ADSCs were treated with 5%, 15% and 25% HQT-containing serum diluted in culture medium for 48 h, respectively. To block exosome generation and release, ADSCs were treated with exosome inhibitor 10µM GW4869 (#S7609, Selleck, Houston, TX, USA) for 2 h prior to treatment of HQT-containing serum. Moreover, MH7A cells were exposed to 1 µg/mL LPS (#L2880, Sigma, St. Louis, MO, USA) for 24 h to establish RA cell model.

### Co-culture of MH7A cells with ADSCs

Co-culture of MH7A cells with ADSCs was performed using a 12-well Transwell filter-plate (#CLS3460, Corning, Corning, NY, USA). Briefly, 2 × 10^5^/mL MH7A cells were seeded at the bottom well containing DMEM while 2 × 10^5^/mL ADSCs were seeded at the upper pore polyester membrane insert (0.4 μm pore size) containing Human Adipose-derived Stem Cell Complete Culture Medium. Then cells were co-cultured for 48 h.

### Quantitative reverse transcription-PCR (qRT‒PCR)

Total RNA was extracted from cells or exosomes by TRIzol reagent (#15596026, Invitrogen, Carlsbad, CA, USA). For miRNAs, first-strand cDNA synthesis and qRT-PCR were performed using All-in-One™ miRNA qRT-PCR Detection Kit (#AOMD-Q050, GeneCopoeia, Beijing, China) based on 7500 Fast Real-Time PCR System (Applied Biosystems, Foster City, CA, USA). For mRNAs, first-strand cDNA was synthetized by PrimeScript II 1st Strand cDNA Synthesis Kit (#6210A, Takara, Dalian, Liaoning, China) while qRT-PCR was performed using TB Green Premix Ex Taq™ (Tli RNaseH Plus) (#RR420A, Takara) based on 7500 Fast Real-Time PCR System (Applied Biosystems). Then the amount of target genes was analyzed using 2^−ΔΔCt^ method [23], with U6 for miRNAs or GAPDH for mRNAs as the internal reference as previously described [10]. The primers used in the current study were listed in Table 1.

**Table 1:** Sequences of primers used for qRT-PCR

Genes		Sequences (5’-3’)	Product (bp)
miR-125b-5p	Forward	TCCCTGAGACCCTAACTTGTGA	22
	Reverse	Universal primer from All-in-One™ miRNA qRT-PCR Detection Kit	
CD63	Forward	GGGCTGCTAACTACACAGATT	125
	Reverse	CTTATGGATCGCCTTCTCGTT	
U6	Forward	CTCGCTTCGGCAGCACA	94
	Reverse	AACGCTTCACGAATTTGCGT	
GAPDH	Forward	AACGGATTTGGTCGTATTGGG	211
	Reverse	CCTGGAAGATGGTGATGGGAT	

### Cell counting kit-8 (CCK-8) assay

In brief, 1 × 10^4^/well ADSCs were collected and seeded into a 96-well plate (#PPP-001-030, Bestopbio, Beijing, China) at 1, 2, 3 day(s) after the treatment of HQT-containing serum, and then 10µL CCK-8 solution (#C0037, Beyotime, Shanghai, China) was added to incubate ADSCs for 4 hours at 37℃. Next, the absorbance at 450 nm was detected utilized a microplate reader (Multiscan MK3, Thermo Fisher Scientific, Cleveland, OH, USA) as previously described [24].

### Flow cytometric analysis for cell apoptosis

ADSCs were collected and washed twice with PBS followed by the re suspension into 100µL incubation buffer (10 mM HEPES/NaOH, 140 mmol/L NaCl, 5 mM CaCl_2_). Then ADSCs were incubated with 10µL Annexin V-FITC (#KGA106, Keygen, Shanghai, China) at RT for 15 min and subsequent moderate propidium iodide (#KGA106, Keygen) at RT for 10 min. Next, ADSCs were analyzed by flow cytometry (BD Calibur, BD Biosciences, San Jose, CA, USA) as previously described [25].

### Isolation and analysis of ADSC-derived exosomes

ExoEasy Maxi Kit (#76064, QIAGEN, Hilden, Germany) was used to isolate exosomes from culture medium of ADSCs according to the exoRNeasy Serum/Plasma Handbook. Then the size of exosomes was identified by a nanoparticle tracking analyzer (N30E, NanoFCM, Nottingham, UK). Moreover, the markers of exosomes were detected by Western Blot using CD9 antibody (1:1000, #bs-5660R, Bioss, Beijing, China) and CD81 antibody (1:1000, #bs-2489R, Bioss).

### Exosome uptake

To detect exosome uptake into MH7A cells, 10 µg of ADSC-derived exosomes were suspended and stained using 100µM PKH26 (#UR52302, Umibio, Shanghai, China) at 37 °C for 15 min, and then centrifuged at 100,000×g at 4 °C for 70 min to remove unbound probes. After the incubation with PKH26-stained ADSC-derived exosomes, the fluorescence signals of PKH26 in MH7A cells were reported by a fluorescence microscopy.

### Enzyme-linked immunosorbent assay (ELISA)

Levels of IL-1β, IL-6 and TNF-α in cellular supernatant of MH7A cells and rat serum were measured by ELISA as previously described [10].

### Western blot (WB)

Total proteins were extracted from cells using RIPA Lysis Buffer (#89900, Thermo Fisher Scientific). Then equal amount of protein from different groups were separated by SDS-polyacrylamide gel electrophoresis (SDS-PAGE) followed by the transfer onto PCDF membranes (0.45 μm pore size, # IPFL00010, Millipore, Bedford, MA, USA). After being blocked with 5% non-fat milk for 1 h at RT, membranes were then incubated with primary antibodies at 4 °C overnight. The next day membranes were washed and incubated with appropriate secondary antibodies for 1 h at RT, membranes were extensively washed. Finally, signals of targeted proteins were detected by BeyoECL Moon Kit (#P0018F, Beyotime). The primary antibodies used in this study were listed as follow: CD9 antibody (1:1000, #bs-5660R, Bioss), CD81 antibody (1:1000, #bs-2489R, Bioss), CD63 antibody (1:1000, #bs-1523R, Bioss), NF-κB p65 antibody (1:1000, #bs-5660R, Bioss) and GAPDH antibody (1:10000, #KC-5G5, Aksomics, Shanghai, China).

### Knockdown of miR-125b-5p and CD63 in ADSCs

First, short hairpin RNA (shRNA) against pre-miR-125b or CD63 was cloned into pLOV-CMV vector. Then 15 µg pLOV-CMV vector, 5 µg pMDL vector (envelop vector) and 10 µg psPAX2 vector (packaging vector) were mixed and transfected into 293T cells cultured in DMEM using Lipofectamine 2000 (Invitrogen). Viral supernatant was collected, filtered and concentrated at 3th day after transfection. Next, 1mL viral supernatant was used to infect ADSCs for 48 h. Finally, knockdown of miR-125b-5p and CD63 was confirmed by qRT-PCR.

### Luciferase reporter assay

Luciferase reporter assay was performed as previously described [10]. Briefly, the *CD63* gene promoter was cloned into pmirGLO luciferase reporter vector (Promega, Madison, WI, USA) followed by the transfection into MH7A cells using Lipofectamine 2000 (Invitrogen). Next, relative luciferase activity was detected by Dual-Luciferase Reporter Assay System (#E1910, Promega) at 48 h post transfection, with Renilla luciferase activity as the internal control.

### Cycloheximide treatment

When grown to approximately 80% confluence, ADSCs were treated with 50 µg/mL cycloheximide (CHX) (#IC0720, Solarbio, Beijing, China) and HQT-containing serum for 3 h, 6 h and 12 h, respectively. Then ADSCs were washed and collected followed by the detection of CD63 levels using Western blot.

### Establish of RA rat model

All animal procedures in this study were performed according to National Institutes of Health guidelines and approved by the Ethics Committee of The Second Affiliated Hospital of Guangzhou University of Chinese Medicine. 25 male 8-week-old Wistar rats purchased from Model Animal Research Center of Nanjing University (China) were randomly divided into control group, RA group, RA + Exosome (Blank) group, RA + Exosome (HQT) group and RA + miR-125b-5p si-Exosome (HQT) group, 5 rats per group. The RA rat model in RA group was established as previously described using collagen-induced arthritis (CIA) model [10]. Besides, rats of RA + Exosome (Blank) group were injected 2 × 10^9^ exosomes derived from ADSCs treated with blank serum into joint, and rats of RA + Exosome (HQT) group were injected 2 × 10^9^ exosomes derived from ADSCs treated with HQT-containing serum into joint, while rats of RA + miR-125b-5p si-Exosome (HQT) group were injected 2 × 10^9^ exosomes derived from miR-125b-5p silenced-ADSCs treated with HQT-containing serum into joint. Exosomes were diluted into physiological saline and injected once per 3 days for 30days. In addition, rats of negative control group were injected with equal volume of physiological saline. After 30 days, arthritis index (AI) was evaluated based on the degree of joint redness and swelling as previously described [10]. Meanwhile, blood was collected and used for subsequent ELISA analysis. Then rats were euthanatized. A flow chart to show all the procedures done within the current study is present in Supplementary Figure S1.

### Statistical analyses

All experiments were repeated three times. Statistical analyses for data presented as mean ± standard error of the mean (SEM) was performed by SPSS 21.0 (IBM Corp., Armonk, NY, USA). Comparison of continuous variables between two groups was carried out using the Mann-Whitney U test and Student’s *t* tests. Moreover, post-hoc Tukey’s test following One way ANOVA was utilized for the statistics among multiple groups. P value < 0.05 was considered statistically significant.

## Results

### HQT elevates LPS-reduced miR-125b-5p level in FLSs by exosomes derived from ADSCs

**Fig. 1:**
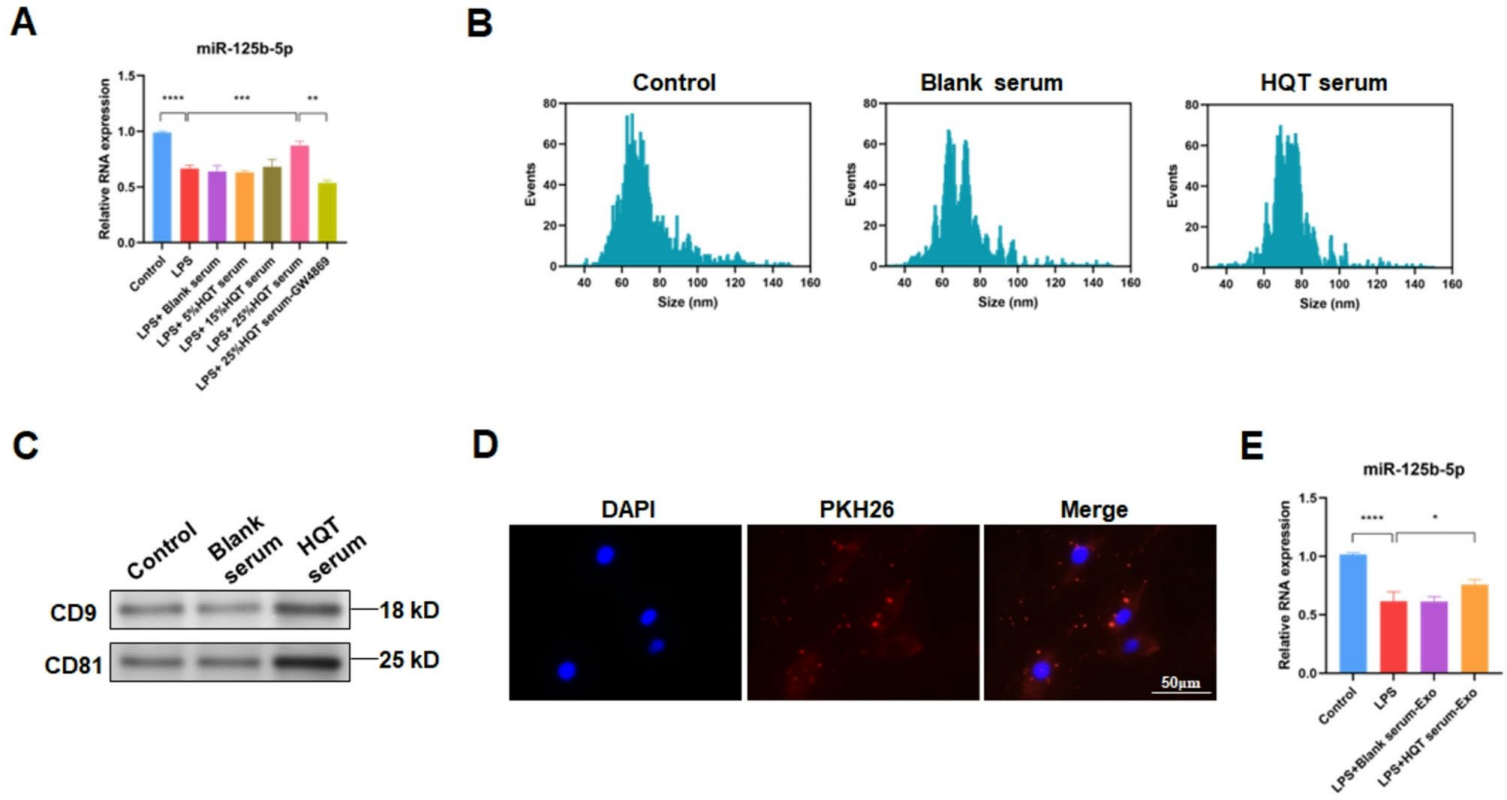
HQT improves LPS-reduced miR-125b-5p level in FLSs by ADSC-derived exosomes. **A** miR-125b-5p level detected in control, LPS-treated, LPS-treated plus co-cultured with ADSCs incubated with blank serum, 5%/15%/25% HQT-containing serum or GW4869 MH7A cells. **B** The size of exosomes derived from ADSC treated with or without blank serum and 25% HQT-containing serum identified by nanoparticle tracking analysis. **C** WB analysis of exosomal protein markers CD63 and CD81 in exosomes derived from ADSC treated with or without blank serum and 25% HQT-containing serum. **D** Representative images of MH7A cells co-cultured with PKH26-stained ADSC-derived exosomes. **E** miR-125b-5p level detected in MH7A cells treated with or without LPS plus exosomes derived from ADSC treated with blank serum or 25% HQT-containing serum. HQT: Huayu-Qiangshen-Tongbi decoction; Exo: exosomes. *P < 0.05, ***P < 0.001, ****P < 0.0001

**Fig. 2:**
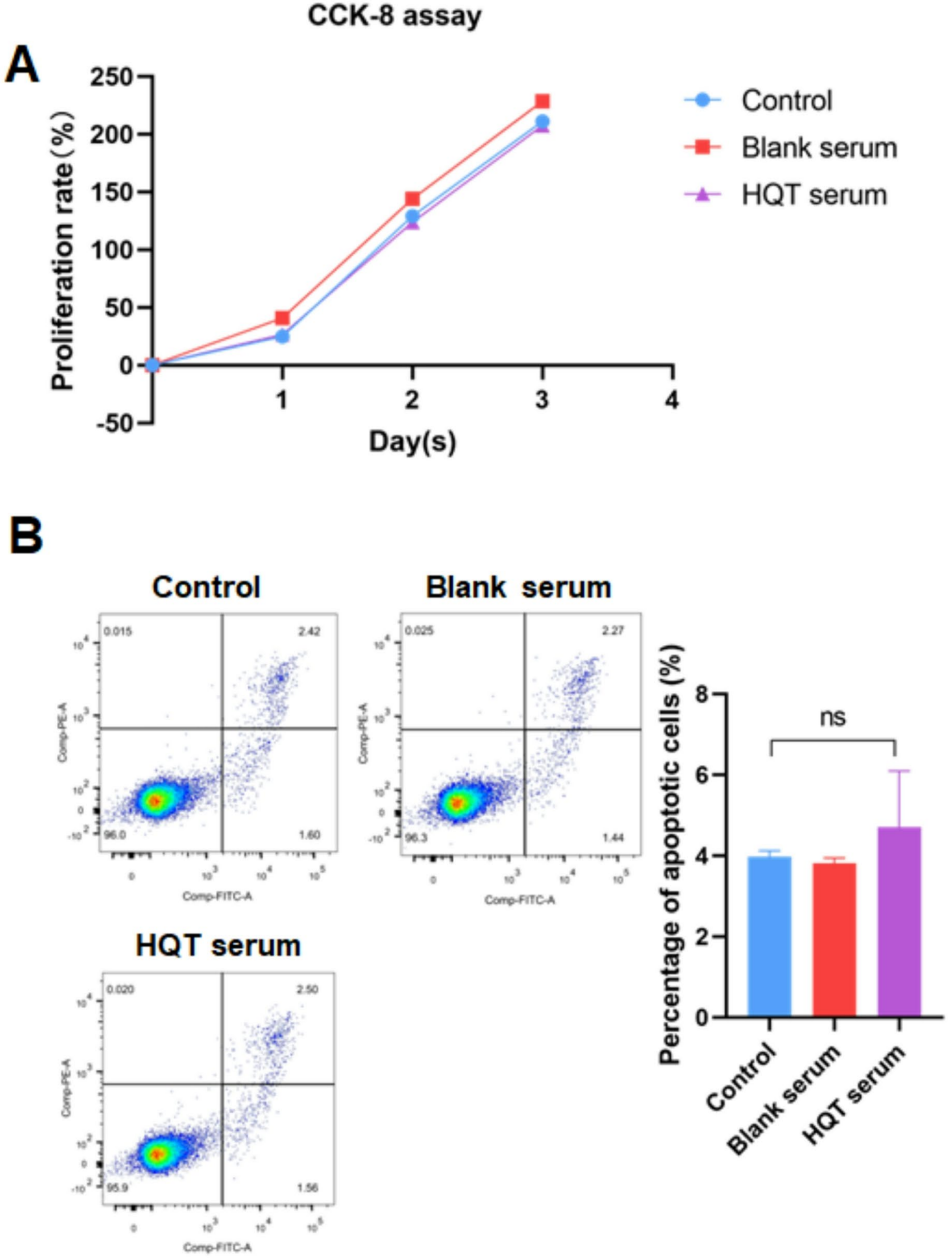
HQT-containing serum has no toxic effect on ADSC. **A** Proliferation rates of ADSCs treated with or without blank serum and 25% HQT-containing serum. **B** Representative images and quantification of flow cytometric apoptosis assay in ADSCs treated with or without blank serum and 25% HQT-containing serum. HQT: Huayu-Qiangshen-Tongbi decoction; ns: no significance

First, HQT-containing serum was generated. Subsequently, the miR-125b-5p level in MH7A cells was deteced by qRT-PCR. Results showed that LPS treatment reduced the miR-125b-5p level in MH7A cells (*P* < 0.0001)(Fig. 1A). Besides, co-culture with ADSCs could not increase LPS-reduced miR-125b-5p level in MH7A cells, but co-culture with ADSCs treated with 25% HQT-containing serum elevated LPS-reduced miR-125b-5p level in MH7A cells (*P* < 0.001) (Fig. 1A). Moreover, treatment of exosome inhibitor (GW4869) reversed the effect of co-culture with ADSCs treated with 25% HQT-containing serum on LPS-reduced miR-125b-5p level in MH7A cells (*P* < 0.01) (Fig. 1A), suggesting HQT should elevate LPS-reduced miR-125b-5p level in FLSs by ADSC-derived exosomes. Furthermore, blank rat serum had no effect on miR-125b-5p level in MH7A cells (Fig. 1A) and 25% HQT-containing serum was utilized in subsequent experiments. In addition, results of CCK-8 and apoptosis assay indicated that treatment of 25% HQT-containing serum did not affect proliferation and apoptosis of ADSCs, indicating that HQT-containing serum had no toxic effect on ADSCs (Fig. 2A and B).

To determine whether HQT regulated miR-125b-5p level of MH7A cells by exosomes derived from ADSCs, ADSC-derived exosomes were isolated after the treatment with or without HQT-containing serum. Analysis revealed that most microvesicles derived from ADSCs treated with or without blank rat serum or HQT-containing serum had a size of 50-200nM detected by NanoSight (Fig. 1B). Besides, exosome markers CD63 and CD81 were found in microvesicles derived from ADSCs treated with or without blank serum or HQT-containing serum (Fig. 1C). These results suggested that ADSC-derived exosomes were isolated successfully.

Next, ADSC-derived exosomes were stained with PKH26 followed by the co-culture with MH7A cells. Then the red fluorescence of PKH26 was observed in MH7A cells, revealing the ADSC-derived exosome uptake into MH7A cells (Fig. 1D). Subsequently, the miR-125b-5p level in MH7A cells was deteced followed by the co-culutre with exosomes derived from ADSCs. Results found that co-cluture with exosomes derived from ADSCs treated with HQT-containing serum elevated LPS-reduced miR-125b-5p level in MH7A cells (*P* < 0.05), whereas co-cluture with exosomes derived from ADSCs treated with blank serum had no effect on LPS-reduced miR-125b-5p level in MH7A cells (Fig. 1E). Above results together suggest that HQT elevates LPS-reduced miR-125b-5p level in FLSs by ADSC-derived exosomes.

### HQT attenuates LPS-induced inflammation in FLSs by exosomal miR-125b-5p derived from ADSCs

**Fig. 3:**
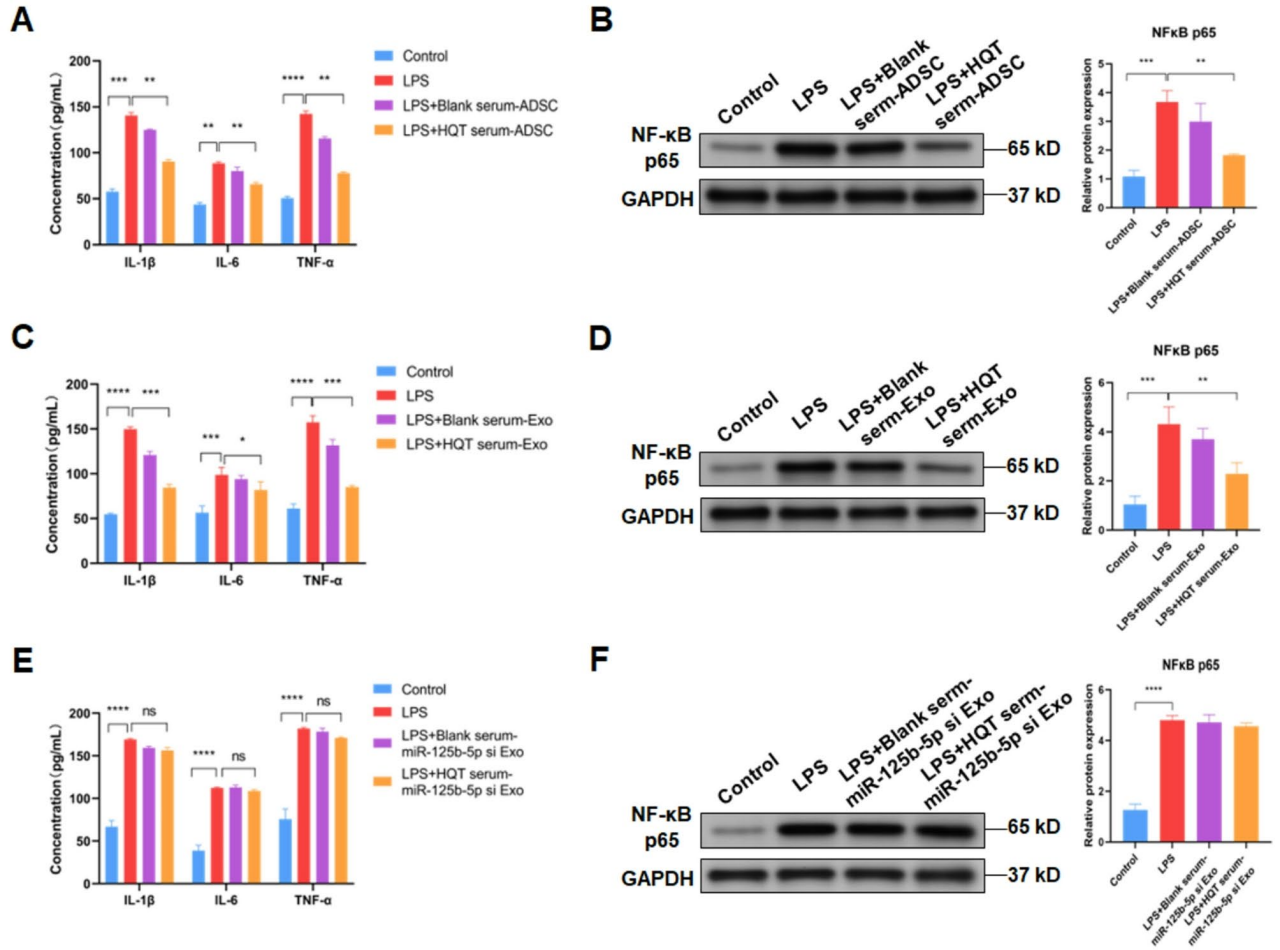
HQT alleviates LPS-induced inflammation in FLSs by exosomal miR-125b-5p derived from ADSCs. **A** Levels of released IL-1β, IL-6 and TNF-α in control, LPS-treated, LPS-treated plus co-cultured with blank serum-treated ADSCs and LPS-treated plus co-cultured with 25% HQT-containing serum-treated ADSCs MH7A cells. **B** The NF-κB p65 level in control, LPS-treated, LPS-treated plus co-cultured with blank serum-treated ADSCs and LPS-treated plus co-cultured with 5% HQT-containing serum-treated ADSCs MH7A cells. **C** Levels of released IL-1β, IL-6 and TNF-α in control, LPS-treated, LPS-treated plus co-cultured with exosomes derived from blank serum-treated ADSCs and LPS-treated plus co-cultured with exosomes derived from 25% HQT-containing serum-treated ADSCs MH7A cells. **D** The NF-κB p65 level in control, LPS-treated, LPS-treated plus co-cultured with exosomes derived from blank serum-treated ADSCs and LPS-treated plus co-cultured with exosomes derived from 25% HQT-containing serum-treated ADSCs MH7A cells. **E** Levels of released IL-1β, IL-6 and TNF-α in control, LPS-treated, LPS-treated plus co-cultured with exosomes derived from blank serum-treated miR-125b-5p-silenced ADSCs and LPS-treated plus co-cultured with exosomes derived from 25% HQT-containing serum-treated miR-125b-5p-silenced ADSCs MH7A cells. **F** The NF-κB p65 level in control, LPS-treated, LPS-treated plus co-cultured with exosomes derived from blank serum-treated miR-125b-5p-silenced ADSCs and LPS-treated plus co-cultured with exosomes derived from 25% HQT-containing serum-treated miR-125b-5p-silenced ADSCs MH7A cells. HQT: Huayu-Qiangshen-Tongbi decoction; Exo: exosomes; si: silence; ns: no significance. *P < 0.05, **P < 0.01, ***P < 0.001, ****P < 0.0001

Next, the effects of exosomal miR-125b-5p derived from ADSCs treated with ADSCs on FLSs were explored. Results demonstrated that LPS treatment increased levels of released IL-1β (*P* < 0.001), IL-6 (*P* < 0.01) and TNF-α (*P* < 0.001) of MH7A cells, whereas co-culture with ADSCs treated with HQT-containing serum further decreased LPS-increased levels of released IL-1β, IL-6 and TNF-α of MH7A cells (*P* < 0.01) (Fig. 3A). Co-culture with ADSCs treated with blank rat serum had no significant effect on LPS-increased levels of released IL-1β, IL-6 and TNF-α of MH7A cells (Fig. 3A). As NF-κB activation is critical for productions of IL-1β, IL-6 and TNF-α and our preivous study has revealed the inhibitory effect of HQT on NF-κB activation in FLSs [10], NF-κB activation was also detected. It was indicated that LPS treatment elevated NF-κB p65 level in MH7A cells (*P* < 0.001), yet co-culture with ADSCs treated with HQT-containing serum decreased LPS-increased NF-κB p65 level in MH7A cells (*P* < 0.01) (Fig. 3B). Co-culture with ADSCs treated with blank rat serum had no significant effect on LPS-increased NF-κB p65 level in MH7A cells (Fig. 3B).

Similar to co-culture with ADSCs, co-culutre with exosomes derived from ADSCs treated with HQT-containing serum reduced levels of released IL-1β (*P* < 0.001), IL-6 (*P* < 0.05) and TNF-α (*P* < 0.001) of MH7A cells under LPS condition (Fig. 3C). Besides, co-culutre with exosomes derived from ADSCs treated with HQT-containing serum reduced LPS-increased NF-κB p65 level in MH7A cells (*P* < 0.01) (Fig. 3D). Co-culture with ADSCs treated with blank rat serum had no significant effect on released IL-1β, IL-6 and TNF-α levels and NF-κB p65 level in MH7A cells under LPS condition (Fig. 3C and D).

To further demtermine the role of exosomal miR-125b-5p derived from ADSCs in FLSs, shRNA was utilized to silence miR-125b-5p in ADSCs (*P* < 0.0001) (Supplementary Figure S2A). Then results revealed that co-culutre with exosomes derived from miR-125b-5p -silenced ADSCs could not decrease LPS-increased levels of released IL-1β, IL-6 and TNF-α (Fig. 3E) and NF-κB p65 level in MH7A cells (Fig. 3F). These data suggest that HQT alleviates LPS-induced inflammation in FLSs by exosomal miR-125b-5p derived from ADSCs.

### HQT enhances the secretion of exosomal miR-125b-5p derived from ADSCs under LPS condition

Then the mechanism of HQT regulating exosomal miR-125b-5p was investigated. Results showed that CD63 and CD81 levels in exosomes derived from ADSCs treated with HQT-containing serum were higher than those in exosomes derived from equal ADSCs treated without HQT-containing serum, indicating that HQT stimulated the secretion of ADSC-derived exosomes (Fig. 1C). Besides, blank rat serum had no effect on the secretion of ADSC-derived exosomes (Fig. 1C).

**Fig. 4:**
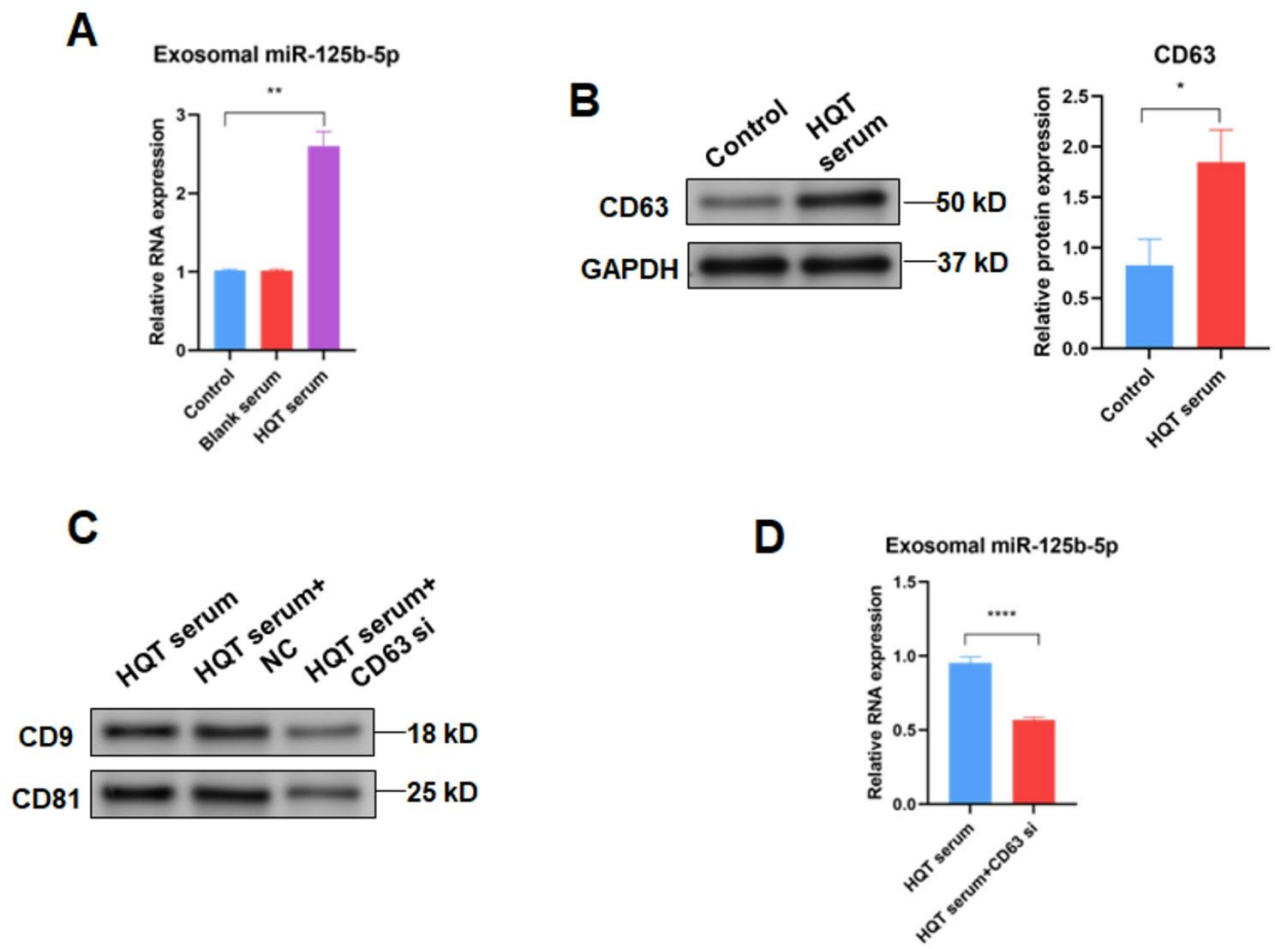
HQT enhances the release of exosomal miR-125b-5p derived from ADSCs by CD63. **A** miR-125b-5p level detected in exosomes derived from control, blank serum-treated and 25% HQT-containing serum-treated ADSCs. **B** CD63 protein level detected in control and 25% HQT-containing serum-treated ADSCs. **C** CD63 protein level detected in 25% HQT-containing serum-treated ADSCs transfected with shRNA NC or CD63 shRNA. **D** miR-125b-5p level detected in exosomes derived from 25% HQT-containing serum-treated ADSCs transfected with or without CD63 shRNA. HQT: Huayu-Qiangshen-Tongbi decoction; NC: negative control; si: silence. *P < 0.05, **P < 0.01, ****P < 0.0001

In addition, the level of exosomal miR-125b from ADSCs was also detected. Results showed that the level of exosomal miR-125b from ADSCs treated with HQT-containing serum was higher than that in exosomes derived from ADSCs treated without HQT-containing serum (*P* < 0.01) (Fig. 4A). Furthermore, blank rat serum had no effect on the level of exosomal miR-125b from ADSCs. Thus, these results suggest that HQT enhances the secretion of exosomal miR-125b-5p derived from ADSCs.

### HQT facilitates the release of exosomal miR-125b-5p derived from ADSCs by CD63

**Fig. 5:**
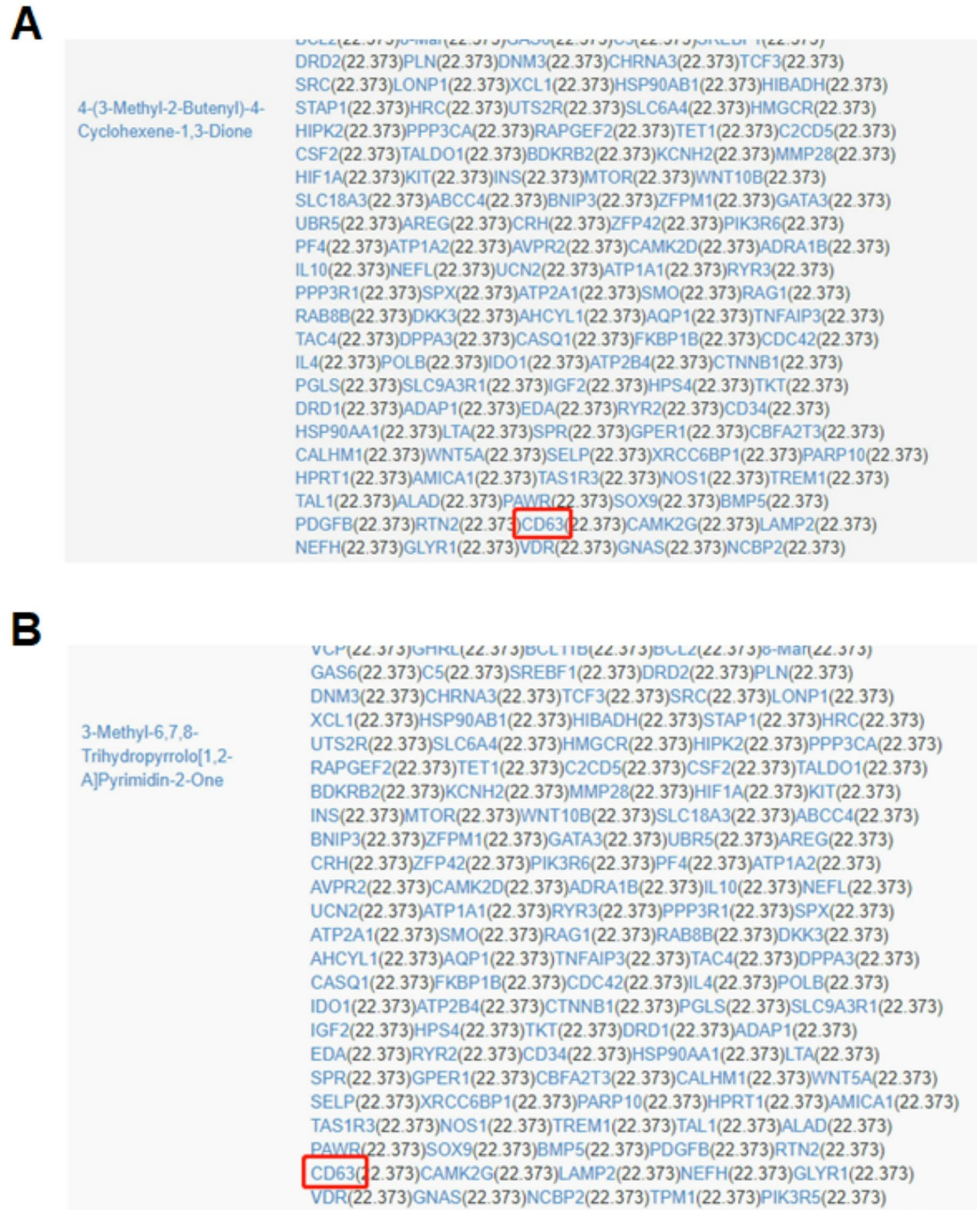
CD63 is the target of HQT. Network pharmacology-based analysis indicated that CD63 should be the target of Radix Dipsaci Asperoidis (Chuanxuduan) (**A**) and Glycyrrhizae Radix et Rhizoma (Gancao) (**B**)

HQT is composed of total 10 herbs, including Salvia miltiorrhiza (Danshen), Dioscorea nipponica Makino (Chuanshanlong), Astragalus membranaceus (Huangqi), Paeonia lactiflora Pall (Baishao), Saussurea involucrate (Tianshanxuelian), Eucommia ulmoides Oliver (Duzhong), Rhizoma Drynariae (Gusuibu), Radix Dipsaci Asperoidis (Chuanxuduan), Radix Rehmanniae Proeparata (Shudi) and Glycyrrhizae Radix et Rhizoma (Gancao) [10]. Network pharmacology-based analysis indicated that CD63 should be the target of Radix Dipsaci Asperoidis (Chuanxuduan) and Glycyrrhizae Radix et Rhizoma (Gancao) (Fig. 5A and B). Results of WB demonstrated that the treatment of HQT-containing serum significantly increased CD63 level in ADSCs (*P* < 0.05) (Fig. 4B). Next, shRNA was utilized to silence CD63 in ADSCs (*P* < 0.001) (Supplementary Figure S2B) followed by the treatment HQT-containing serum. Results showed that exosome markers CD9 and CD81 levels in exosomes derived from CD63-silenced ADSCs treated with HQT-containing serum were lower than those in equal exosomes derived from ADSCs treated with HQT-containing serum in equal protein amounts (Fig. 4C). Moreover, results of qRT-PCR indicated that exosomal miR-125b-5p level derived from CD63-silenced ADSCs treated with HQT-containing serum were lower than that derived from ADSCs treated with HQT-containing serum (*P* < 0.0001) (Fig. 4D). These results suggest that HQT facilitates the release of exosomal miR-125b-5p derived from ADSCs by CD63.

### HQT suppresses CD63 degradation in ADSCs

**Fig. 6:**
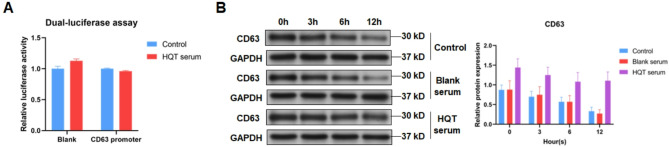
HQT suppresses CD63 degradation in ADSCs. **A** Transcription activity of *CD63* gene was analyzed by relative luciferase reporter activity assay in control and 25% HQT-containing serum-treated ADSCs. **B** CD63 protein level detected in control, blank serum-treated and 25% HQT-containing serum-treated ADSCs after CHX treatment. HQT: Huayu-Qiangshen-Tongbi decoction

Subsequently, the mechanism of HQT regulating CD63 in ADSCs was explored. First, the *CD63* gene promoter was inserted into luciferase reporter gene vector followed by the transfection into ADSCs. Then results of luciferase reporter assay revealed that the luciferase activity of ADSCs treated with HQT-containing serum was consistent with that of ADSCs treated with blank rat serum (Fig. 6A), suggesting HQT had no effect on *CD63* gene transcription.

Next, the CD63 protein degradation in ADSCs was identified after the expose to 50 µg/mL CHX for increasing times. In untreated ADSCs, CD63 began to disappear at 3 h after CHX treatment while there was only 38% protein remained at 12 h after CHX treatment (Fig. 6B). In ADSCs treated with HQT-containing serum, CD63 also began to disappear at 3 h after CHX treatment yet there was 77% protein remained at 12 h after CHX treatment (Fig. 6B). Besides, the blank rat serum had no significant effect on disappearing CD63 (Fig. 6B). These data reveal that HQT inhibits the disappearance of CD63 protein in ADSCs, which might occur by suppressing protein degradation.

### HQT ameliorates joint injury and inflammation by exosomal miR-125b-5p derived from ADSCs in RA rat model

**Fig. 7:**
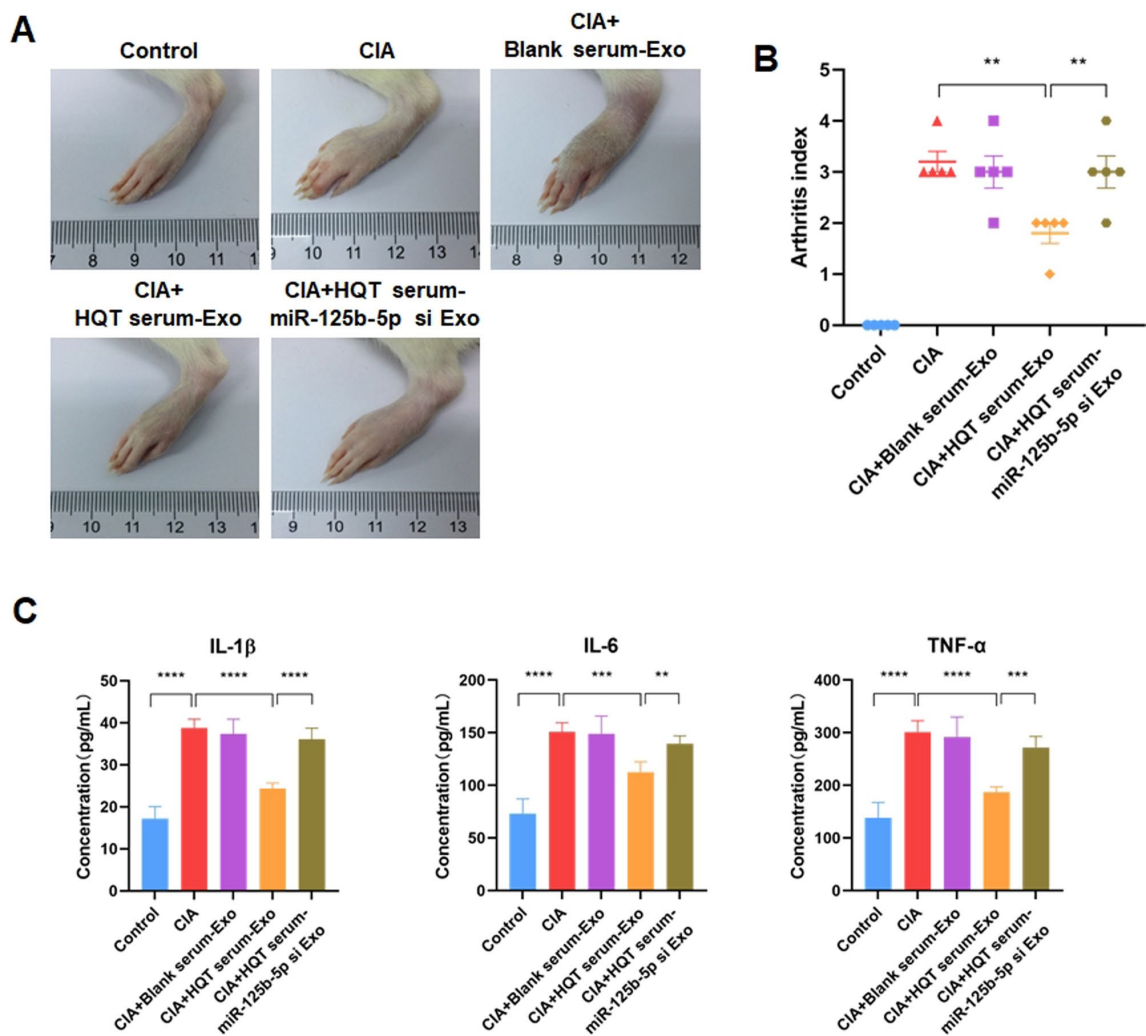
HQT attenuates RA-induced joint injury and inflammation by exosomal miR-125b-5p derived from ADSCs in vivo. **A** Representative image of the joint edema of control, CIA, CIA plus exosomes derived from blank serum-treated ADSCs-injected, CIA plus exosomes derived from 25% HQT-containing serum-treated ADSCs-injected and CIA plus exosomes derived from 25% HQT-containing serum-treated miR-125b-5p-silenced ADSCs-injected rats. **B** The arthritis index (AI) of control, CIA, CIA plus exosomes derived from blank serum-treated ADSCs-injected, CIA plus exosomes derived from 25% HQT-containing serum-treated ADSCs-injected and CIA plus exosomes derived from 25% HQT-containing serum-treated miR-125b-5p-silenced ADSCs-injected rats. **C** Levels of serum IL-1β, IL-6 and TNF-α in control, CIA, CIA plus exosomes derived from blank serum-treated ADSCs-injected, CIA plus exosomes derived from 25% HQT-containing serum-treated ADSCs-injected and CIA plus exosomes derived from 25% HQT-containing serum-treated miR-125b-5p-silenced ADSCs-injected rats. HQT: Huayu-Qiangshen-Tongbi decoction; Exo: exosomes; si: silence. **P < 0.01, ***P < 0.001, ****P < 0.0001

Firstly, RA rat model was established by CIA. It was observed that the joint edema of RA rat model was obviously exacerbated compared to that of rats in control group (Fig. 7A). Besides, injection of exosomes derived from ADSCs treated with 25% HQT-containing serum alleviated the joint edema of RA rat model, but injection of exosomes derived from ADSCs treated with blank rat serum had no significant effect on the joint edema of RA rat model (Fig. 7A). Moreover, injection of exosomes derived from miR-125b-5p-silenced ADSCs treated with 25% HQT-containing serum also had no significant effect on the joint edema of RA rat model (Fig. 7A).

Similarly, the arthritis index (AI) of RA rat model was significantly higher than that of rats in negative control group (*P* < 0.01) (Fig. 7B). Moreover, injection of exosomes derived from ADSCs treated with HQT-containing serum reduced the AI of RA rat model, yet injection of exosomes derived from ADSCs treated with blank rat serum had no significant effect on the AI of RA rat model (*P* < 0.01) (Fig. 7B). In addition, injection of exosomes derived from miR-125b-5p-silenced ADSCs treated with HQT-containing serum had no significant effect on the AI of RA rat model as well (Fig. 7B).

Next, the serum levels of inflammation-related cytokines in rats were detected using ELISA. Results revealed that the serum levels of IL-1β, IL-6 and TNF-α was dramatically increased in RA rat model compared to those in rats of negative control group (*P* < 0.0001) (Fig. 7C). Besides, injection of exosomes derived from ADSCs treated with HQT-containing serum reduced the serum levels of IL-1β (*P* < 0.0001), IL-6 (*P* < 0.001) and TNF-α (*P* < 0.0001) of RA rat model, while injection of exosomes derived from ADSCs treated with blank rat serum had no significant effect on the serum levels of IL-1β, IL-6 and TNF-α of RA rat model (Fig. 7C). Furthermore, injection of exosomes derived from miR-125b-5p-silenced ADSCs treated with HQT-containing serum also had no significant effect on the serum levels of IL-1β (*P* < 0.0001), IL-6 (*P* < 0.01) and TNF-α (*P* < 0.001) of RA rat model (Fig. 7C). Therefore, above data together suggest that HQT ameliorates joint injury and inflammation by exosomal miR-125b-5p derived from ADSCs in RA rat model.

## Discussion

Our results indicate that HQT elevates LPS-reduced miR-125b-5p level in FLSs by exosomes derived from ADSCs via enhancing the secretion of exosomal miR-125b-5p derived from ADSCs. Besides, HQT attenuates inflammation of FLSs in RA by exosomal miR-125b-5p derived from ADSCs in vitro and in vivo. Mechanistically, HQT suppresses CD63 degradation to facilitate the release of exosomal miR-125b-5p derived from ADSCs.

Numerous studies have revealed the crucial role of exosomal miR-125b-5p in human diseases. For instance, exosomal miR-125b-5p derived from mesenchymal stem cells (MSCs) accelerates tubular repair by inhibiting p53 in acute kidney injury [26]. Besides, exosomal miR-125b-5p derived from MSCs improves autophagic flux to alleviate myocardial infarction-induced injury [27]. Furthermore, exosomal miR-125b-5p derived from cancer-associated fibroblasts prohibits the expression of adenomatous polyposis coli (APC) in pancreatic cancer (PC) cells to promote PC progress [28]. Moreover importantly, exosomal miR-125-5p derived from ADSCs suppresses the ferroptosis of pulmonary microvascular endothelial cells to attenuate sepsis-induced lung injury [14]. However, the role of exosomal miR-125-5p in RA has not been revealed. Thus, our study uncovers the role of exosomal miR-125-5p in RA for the first time.

Cytokines including IL-1β, IL-6 and TNF-α play central roles in the pathogenesis of RA [29–31]. The present findings demonstrate that HQT reduces released IL-1β, IL-6 and TNF-α levels by miR-125-5p in FLSs. Previous studies have found that NF-κB facilitates the synthesis of IL-1β, IL-6 and TNF-α in RA cell model [32–34]. As HQT suppresses NF-κB-triggered inflammation in rheumatoid FLSs by miR-125b-5p [10], these studies suggest that HQT decreases released IL-1β, IL-6 and TNF-α levels by inhibiting NF-κB via miR-125-5p in FLSs.

It has been revealed that CD63 interacts with latent membrane protein 1 (LMP1) of Epstein-Barr virus to facilitate the package of LMP1 into exosomes derived from infected cancer cells [35, 36]. To date, these is no evidence indicate that CD63 helps RNAs including miRNAs sort into exosomes. Our data found that CD63 elevated exosomal miR-125b-5p derived from ADSCs. As RNA binding proteins (RBPs) are essential for the package of miRNAs into exosomes [37, 38], it is suggested that CD63 might facilitate the package of miR-125b-5p into exosomes derived from ADSC by binding with RBPs.

Results of this study demonstrate that HQT could suppress CD63 degradation in ADSCs. Some studies have revealed the effects of TCM on protein degradation. It has been reported that daidzein prevents skeletal muscle protein degradation to ameliorate cisplatin-induced muscle atrophy [39]. Besides, solasonine extracted from *Solanum nigrum L.* facilitates solute carrier family 7 member 11 (SLC7A11) degradation to induce ferroptosis of pancreatic cancer cells [40]. More importantly, TCM formula Xiaofeng granules suppresses proteoglycan degradation in chondrocytes to attenuate gouty arthritis [41]. Therefore, our findings expand the knowledge of the effects of TCM on protein degradation in arthritis, which might provide novel insight in RA treatment.

Ubiquitin-proteasome system (UPS) and autophagy are considered as the major protein degradation systems [42]. A previous study has found that berberine from a set of TCM induces programmed cell death ligand-1 (PD-L1) degradation via UPS to activate antitumor immunity [43]. Besides, Catalpol, Puerarin and Daidzei could activate UPS to suppress neuronal death and treat spinocerebellar ataxia type 3 [44]. In addition, numerous studies have indicated that various TCM ameliorate neurodegenerative diseases, glomerular diseases, Alzheimer’s disease and cancers by regulating autophagy [45–49]. Thus, HQT might suppress CD63 degradation through regulating UPS or autophagy in ADSCs.

However, there are some limitations of this study. The current study could be strengthened by investigating the mechanisms of CD63 facilitating exosomal miR-125b-5p package and HQT suppressing CD63 degradation.

## Conclusions

**Fig. 8:**
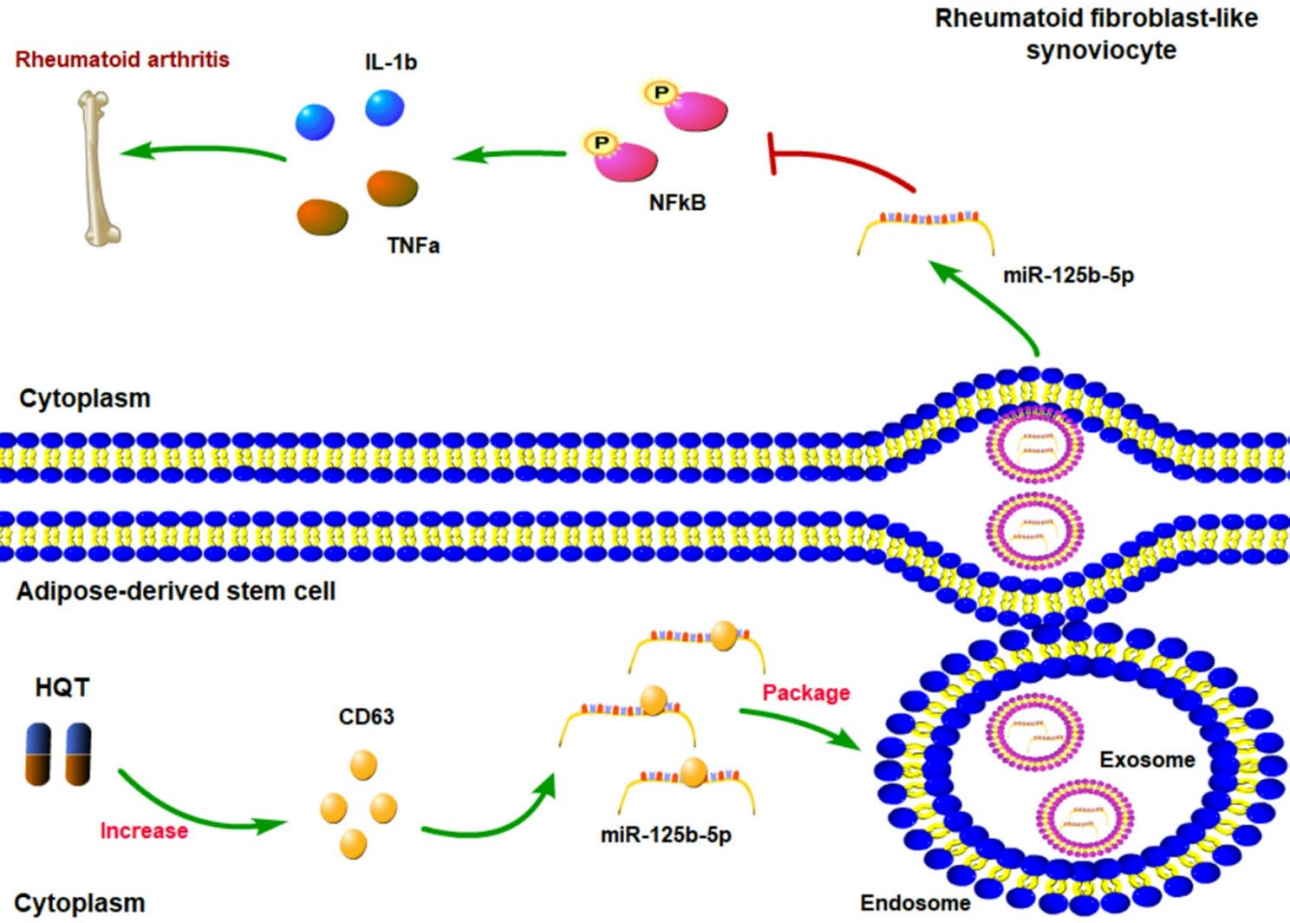
Schematic diagram of molecular mechanisms for the current study. HQT suppressed CD63 degradation to facilitate the release of exosomal miR-125b-5p derived from ADSCs and subsequently exosomal miR-125b-5p was transferred into FLSs to attenuate RA-induced inflammation

In conclusion, the present study indicates that HQT suppresses CD63 degradation to facilitate the release of exosomal miR-125b-5p derived from ADSCs and subsequently exosomal miR-125b-5p is transferred into FLSs to attenuate RA-induced inflammation (Fig. 8). These findings reveal the mechanism of HQT elevating miR-125b-5p expression in FLSs and provide novel therapeutic strategy for RA treatment.

## Electronic supplementary material

Below is the link to the electronic supplementary material.

Supplementary Material 1

Supplementary Material 2

## Data Availability

All data utilized in this work could be obtained from the corresponding author upon request.
