# Supplementary material for: Chinese herbal formula Huayu-Qiangshen-Tongbi decoction ameliorates rheumatoid arthritis through enhancing the release of exosomal miR-125b-5p derived from adipose-derived stem cells by CD63
Source: Biol Res. 2025 Jul 10;58:47. doi: 10.1186/s40659-025-00628-z (PMC12243140; doi:10.1186/s40659-025-00628-z)

**Supplementary Figure S1 A flow chart to show all the procedures done within the current study.**

**Supplementary Figure S2 Efficiency of shRNAs. A** miR-125b-5p level detected in control and shRNA-transfected MH7A cells. **B** CD63 mRNA level detected in control and shRNA-transfected ADSCs.

**Supplementary Figure S1**


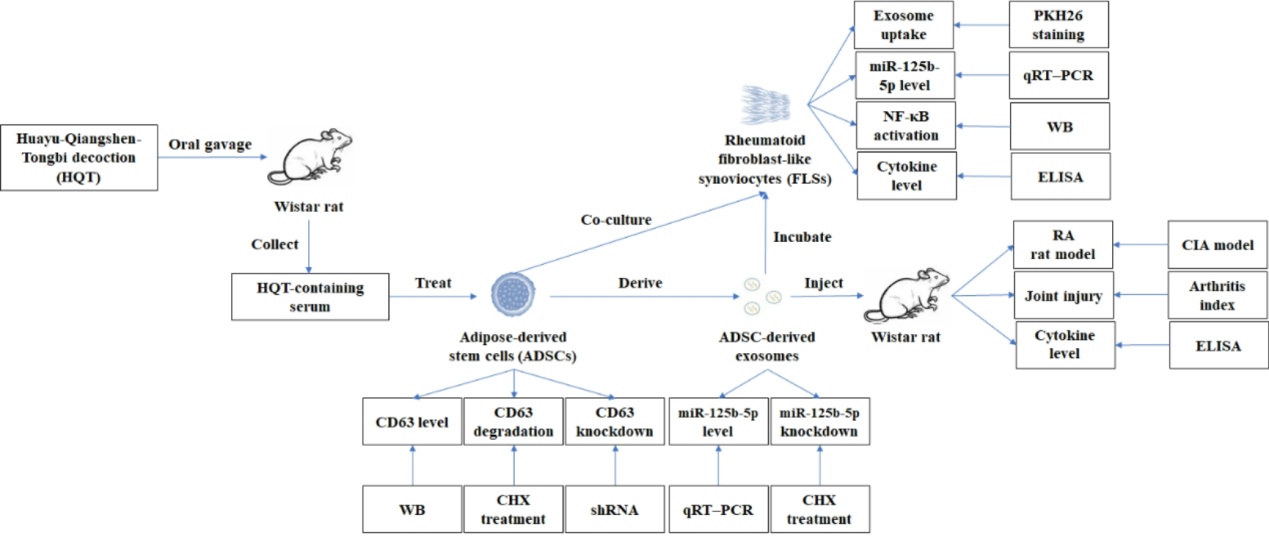


**Supplementary Figure S2**


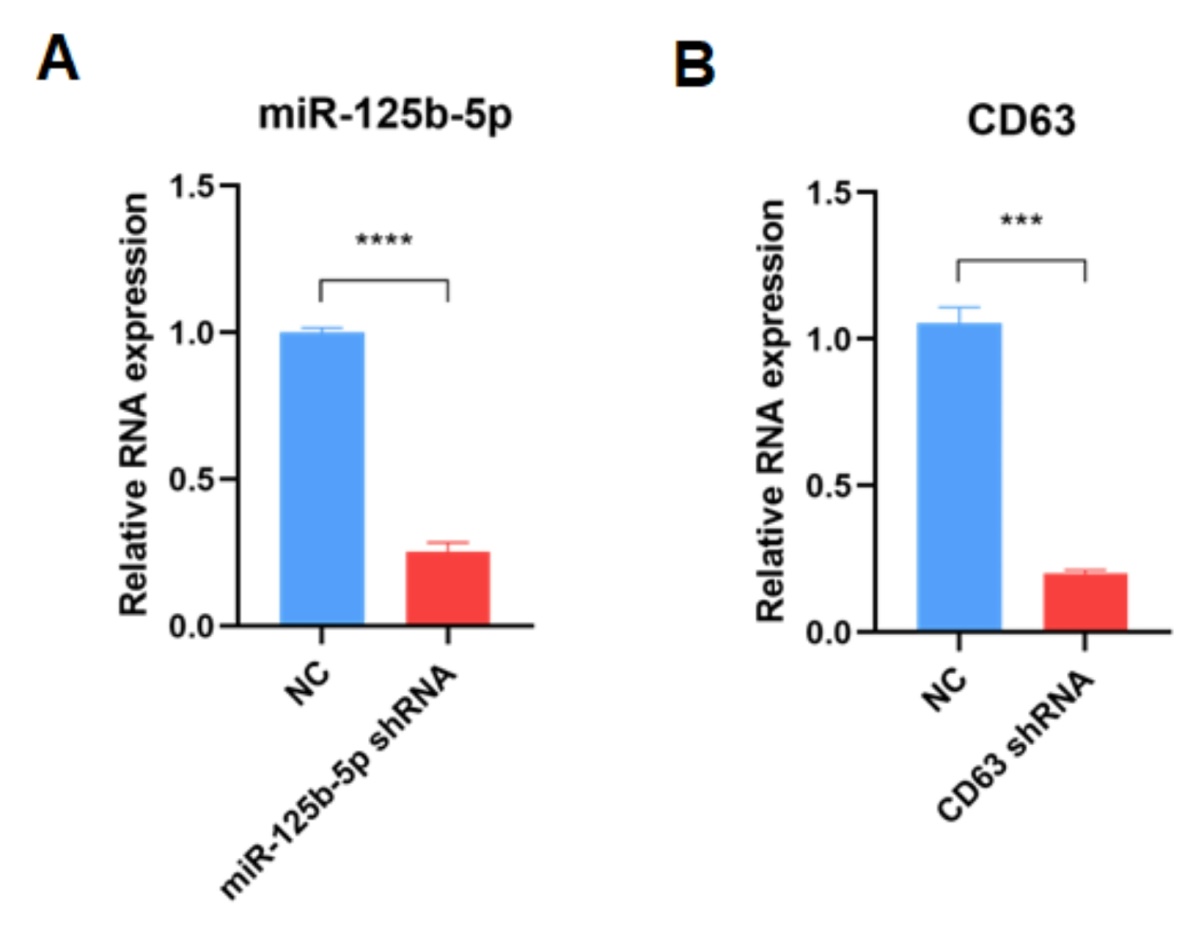

Supplement: Supplementary file 2 — Supplementary Material 2 [file 40659_2025_628_MOESM2_ESM.docx]
